# What Are SAVR Indications in the TAVI Era?

**DOI:** 10.3390/jcm14072357

**Published:** 2025-03-29

**Authors:** William Davalan, Walid Ben Ali, Sebastián Mrad, Pierre-Emmanuel Noly

**Affiliations:** 1 Montreal Heart Institute Research Centre, Université de Montréal, 5000 Est Belanger Street, Montreal, QC H1T 1C8, Canada; 2 Faculty of Medicine, McGill University, Montreal, QC H3A 0G4, Canada; 3 Department of Cardiac Surgery, Montreal Heart Institute, Université de Montréal, Montreal, QC H1T 1C8, Canada; 4 Department of Interventional Cardiology, Montreal Heart Institute, Université de Montréal, Montreal, QC H1T 1C8, Canada

**Keywords:** surgical aortic valve replacement—SAVR, transcatheter aortic valve implantation—TAVI, aortic valve disease, lifetime management

## Abstract

While surgical aortic valve replacement (SAVR) has traditionally been regarded as the gold standard for severe symptomatic aortic stenosis (AS), transcatheter aortic valve implantation (TAVI) has emerged as a compelling less invasive alternative for patients with severe AS across the entire surgical risk spectrum. Despite TAVI’s increasing utilization and promising outcomes, SAVR continues to be an essential treatment modality for certain patient populations, including individuals with complex aortic anatomies unsuitable for TAVI, patients presenting with significant aortic regurgitation, individuals requiring concomitant surgical procedures, and cases involving infective endocarditis. Furthermore, concerns regarding the long-term durability and complication profile of transcatheter valves underscore the importance of individualized patient assessment, especially for younger patients requiring optimal lifetime management strategies. This review examines the evolving role of SAVR amidst the growing adoption of TAVI and highlights key considerations for selecting the most appropriate treatment strategy for patients with aortic valve disease, incorporating insights from recent advancements in transcatheter technologies and the latest clinical trial evidence.

## 1. Introduction

Aortic stenosis (AS) is the most prevalent form of valvular heart disease [1], affecting approximately 2–5% of individuals over the age of 65 [2]. The prevalence of AS is expected to rise as the population ages, given that the incidence of AS increases with age. Severe symptomatic AS carries a poor prognosis in the absence of valve replacement, with 1- and 5-year mortality rates of 40–50% [3] and 94% [4], respectively. Surgical aortic valve replacement (SAVR) has been regarded traditionally as the gold standard for treating severe symptomatic AS [5]. However, over the past two decades, transcatheter aortic valve implantation (TAVI) has emerged as a minimally invasive alternative, initially for high- and intermediate-risk surgical patients and more recently for select low-risk patients [6,7,8,9]. TAVI has been rigorously evaluated in a series of randomized clinical trials comparing it directly with SAVR [6,7,8,9,10,11,12], consistently demonstrating superior or non-inferior outcomes for all-cause mortality and stroke over the longest available follow-up periods [13,14,15,16]. Consequently, both American [17,18] and European [19] guidelines now recommend transfemoral TAVI and SAVR as viable options for most patients with severe symptomatic AS. These positive outcomes, although primarily based around studies with limited follow-up periods and selected population, have significantly shifted the management paradigm for AS, establishing TAVI as a less invasive treatment option that enables quicker recovery while offering comparable clinical benefits to SAVR.

However, while TAVI has shown promising outcomes across broad patient populations, concerns persist regarding its efficacy in patients with complex anatomies. Additionally, concerns remain about the long-term durability of transcatheter heart valves (THV) and the implications of transcatheter procedures for lifetime management. As eligibility criteria for TAVI continue to expand, including younger low-risk patients with longer life expectancies, the potential need for subsequent reinterventions due to structural valve deterioration becomes a critical consideration starting at the index procedure. Therefore, choosing between TAVI and SAVR should involve a personalized evaluation by a multidisciplinary heart team, taking into account patient-specific characteristics to determine the optimal treatment strategy (Table 1).

This review aims to assess the current role of SAVR in aortic stenosis, comparing it with the latest evidence for TAVI by evaluating outcomes in patients with complex anatomies and offering insights into optimal lifetime management strategies.

## 2. Complex Anatomy Unsuitable for Tavi

**Figure 1:**
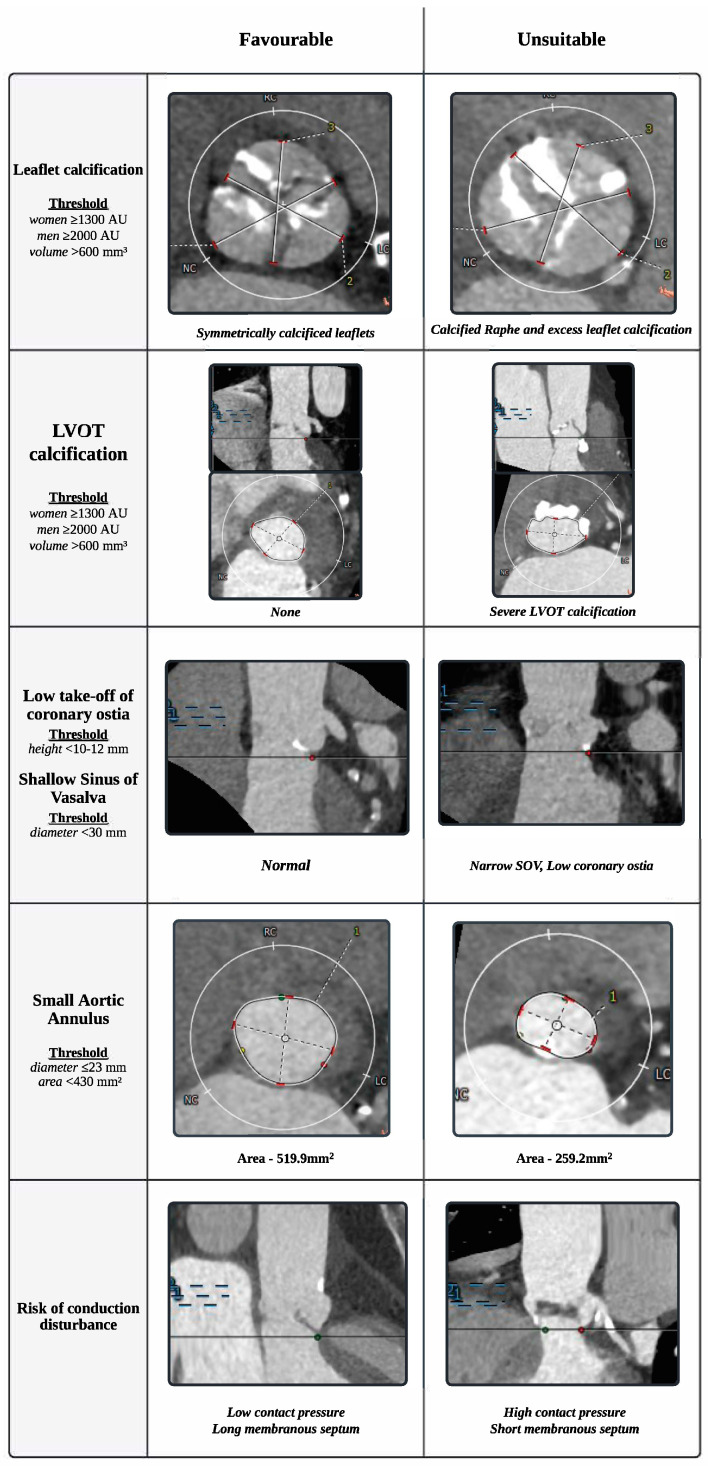
Anatomical Risk Stratification of Native Aortic Valve Morphology for Suitability in Transcatheter Aortic Valve Implantation (TAVI). Abbreviations: LVOT—Left Ventricular Outflow Tract; SOV—Sinus of Vasalva.

The current evidence favoring TAVI over SAVR is largely derived from studies conducted in carefully selected patient groups; however, patients with complex anatomies have been systematically excluded from landmark studies [8,12]. For instance, in the PARTNER_3_ [20] and EvolutLowRisk trials [21], a significant proportion of patients (33% and 15%, respectively) failed the screening process, underscoring potential limitations in generalizing these recommendations for subgroups with specific anatomical complexities. Indeed, anatomical risk stratification continues to play a decisive role in determining the appropriate treatment between SAVR and TAVI, with SAVR being the preferred option for patients who present with anatomical features unfavorable for TAVI (Figure 1), including extensive annular and left ventricular outflow tract (LVOT) calcifications, extreme aortic annulus diameters, low coronary heights, and bicuspid aortic valve. Moreover, SAVR remains the preferred option for patients with extended indications requiring greater procedural versatility, such as isolated aortic regurgitation, the need for additional interventions for concomitant conditions, or inadequate peripheral access. 

### 2.1. Bicuspid Aortic Valve

Bicuspid aortic valve (BAV) disease accounts for 49% of AS patients requiring SAVR [22], but only 5–10% of patients undergoing TAVI [13,14,15,23]. Treating BAV stenosis presents unique challenges due to the distinct anatomical features of BAV, including a heavily calcified raphe and eccentric leaflet calcifications [24,25], which complicate the deployment of THV and favor perivalvular leakage (PVL) or strokes. Furthermore, BAV is commonly associated with concomitant aortopathy, occurring in 20–40% of cases, where predilatation may have a significant impact on the success of TAVI procedures [26,27]. In patients with BAV stenosis requiring valve replacement, elective replacement of the ascending aorta is recommended when the diameter exceeds 45 mm [28,29] or in the presence of other aortopathic features.

**Figure 2:**
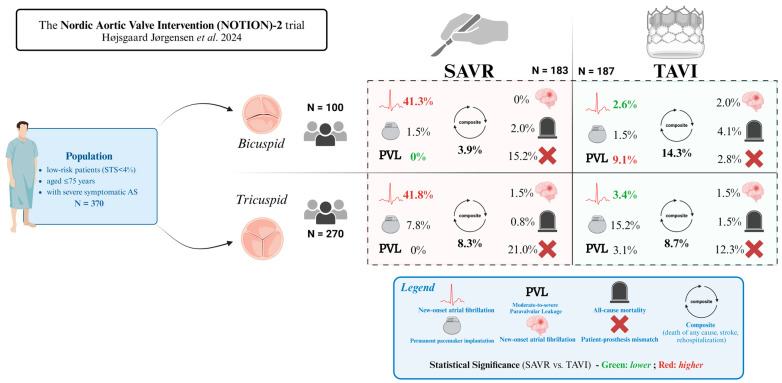
The Nordic Aortic Valve Intervention (*NOTION*)-2 Trial [31]: Surgical Aortic Valve Replacement (SAVR) vs. Transcatheter Aortic Valve Implantation (TAVI) Outcomes in Bicuspid and Tricuspid Valves.

A subgroup analysis of the NOTION-2 trial [28], which enrolled low surgical risk patients aged ≤75 years with severe symptomatic BAV stenosis [30], revealed a higher risk of moderate-or-greater PVL in the TAVI group (9.1% vs. 0%, *p* = 0.04), as well as a trend towards a higher risk of all-cause mortality, stroke, or re-hospitalization in the TAVI group (14.3%) compared to the SAVR group (3.9%), although not statistically significant (*p* = 0.07) (Figure 2). These findings are, however, limited by the scarcity of randomized controlled trials (RCTs) data on the efficacy and safety of TAVI in BAV patients [13,14,15,23]. Further evaluation through dedicated RCTs, such as the forthcoming NAVIGATE bicuspid trial, is necessary to assess the evolving suitability of TAVI for the unique anatomical features of BAV patients. Consequently, SAVR remains the preferred treatment for BAV patients who are at low surgical risk or present with significant aortopathy. Moreover, given the higher prevalence of BAV among younger patients, therapeutic strategies for BAV management must prioritize lifetime considerations, as will be explored in a later section.

### 2.2. Aortic Regurgitation

Similarly, patients with native severe aortic regurgitation (AR) exhibit unique anatomical features, including minimal leaflet calcification, larger aortic annulus dimensions, and increased stroke volume secondary to AR [32]. These factors complicate valve anchoring and positioning during TAVI. In addition, patients with severe AR often present with varying degrees of aortic root dilatation, which may necessitate aortic root replacement [33].

Despite significant advancements in TAVI, its associated outcomes for AR remain inferior to those for AS, with higher rates of complications, such as increased conversion to surgery and moderate-to-severe PVL, as well as a 30-day mortality rate of 5–10% [19,34,35,36,37]. While the PANTHEON trial [38] reported a modest device success rate of 76.8% in patients with pure AR treated with off-label TAVI devices, recent trials [39,40] reported improved preliminary outcomes, with the ALIGN-AR trial [40] reporting a 95% technical success rate, a 30-day mortality rate of 2.2%, and no- or trace-PVL in 92.2% of patients at 12 months. Nevertheless, the pacemaker implantation rate remains high at 24%, underscoring the need for further refinement. A recent systematic review and meta-analysis (2024) [41] of 33,484 patients with AR showed that in-hospital mortality was comparable between TAVI and SAVR (*p* = 0.63), though TAVI was associated with a higher incidence of pacemaker implantation (*p* < 0.001). Of note, TAVI was favored over SAVR regarding in-hospital stroke (*p* < 0.001), in-hospital acute kidney injury (*p* < 0.001), major bleeding (*p* < 0.001) [41]. However, current data are limited to a maximum follow-up period of one year, with most studies focusing on early post-procedural outcomes [41,42].

Therefore, until further large-scale trials establish TAVI’s safety and efficacy for AR, SAVR remains the preferred treatment for low surgical risk patients [17,19,43,44]. Current European Society of Cardiology (ESC)/European Association for Cardio-Thoracic Surgery (EACTS) guidelines provide a Class I recommendation for SAVR in symptomatic patients or those with reduced LVEF. They also recommend pre-emptive SAVR for asymptomatic patients with preserved LVEF in the setting of a dilatated left ventricle, defined by a left ventricular end-systolic diameter (LVESD) >50 mm (Class I indication) or an indexed LVESD >20 mm/m^2^ (Class IIb indication). However, TAVI is currently accepted for patients declined for surgery, considering untreated AR patients have a 2-year mortality rate of 21%.

In cases of severe AR with favorable anatomy, aortic valve repair and valve-sparing aortic root replacement (VSRR) is an effective treatment option that restores competence in regurgitant aortic valves, while preserving avoidance of the risks related to valve replacement. This approach is particularly beneficial for younger patients and those with connective tissue disorders, as it eliminates the risks associated with prosthetic valves, such as thromboembolism, endocarditis, and the requirement for lifelong anticoagulation [45,46]. Recent studies underscore the durability of VSRR, with freedom from reintervention rates of 92.2% at 5 years and 74.1% at 15 years [46]. For patients with AR and bicuspid aortic valves who present unique anatomical challenges, VSRR has still shown favorable outcomes, emphasizing the importance of careful patient selection and surgical expertise in achieving successful results [47].

### 2.3. Annular and Left Ventricular Outflow Tract (LOVT) Calcifications

Expanding on the examination of BAV and AR conditions which are often characterized by abnormal calcification patterns, severe and asymmetrical calcification around the aortic annulus—extending into the LVOT—presents significant challenges for TAVI [48,49,50,51]. Indeed, extensive calcification increases biomechanical stress on the newly implanted valve during its deployment, heightening the risk of annular rupture, which has a mortality rate exceeding 75% [48,51,52]. Furthermore, heterogeneous calcification patterns in the annulus and LVOT can impair the full and symmetrical expansion of the prosthesis, resulting in significant PVL [53]. The quantity, location, and pattern of calcification have been demonstrated as strong predictors of PVL, adversely affecting clinical outcomes after TAVI [54,55], with thresholds of aortic valve calcification (AVC) established at ≥1300 AU in women and ≥2000 AU in men for severe AS [56,57]. SAVR allows for direct visualization and surgical management of calcific deposits, enabling the ability to excise calcifications and directly suture the prosthesis to the aortic annulus. By enabling the removal of calcific deposits and ensuring better valve seating, SAVR mitigates many of the risks associated with TAVI in patients with complex calcification patterns and provides enhanced control over sealing potential regurgitation sites. Moderate-to-severe PVL has a significant impact on survival, with patients experiencing moderate or severe PVL showing a markedly higher risk of overall mortality, as indicated by hazard ratios ranging from 2.18 to 3.02 [58]. The pooled incidence of moderate-to-severe PVL following TAVI is estimated at 11.7% [15,59], whereas mild PVL is considerably more common, occurring in 30–36% of TAVI cases compared to only 3% in SAVR [8,12]. Although mild PVL is often viewed as less clinically significant, it has been linked to a substantially increased risk of all-cause mortality within five years post-TAVI (54.6% vs. 43.8% for patients with none/trace PVL) [60], regardless of baseline clinical or echocardiographic characteristics. Therefore, SAVR may be a more suitable option than TAVI for patients with severe calcification extending into the LVOT, as they are at higher risk for PVL.

Moreover, in addition to excising excessive calcification deposits, SAVR involves the complete removal of the calcified valve, whereas TAVI compresses it against the aortic root upon deployment of the THV. Retained calcification deposits in patients undergoing TAVI can act as a source of embolism both during and after the procedure, elevating the risk of thrombogenesis and potentially resulting in subclinical leaflet thrombosis. Procedural techniques involving wire and catheter manipulation, balloon aortic valvuloplasty, THV positioning, and post-dilation heighten the risk for calcific and atheromatous embolization during TAVI procedures and contribute to periprocedural cerebrovascular events [61,62,63]. The CENTER_2_ [64] trial reported a 30-day stroke incidence of 2.2% following TAVI between 2007 and 2022. Various anticoagulation and antiplatelet strategies, as well as cerebral embolic protection devices (CEPD) have been evaluated through trials [65,66,67], but no significant differences in cerebrovascular event rates were observed [68].

Alternatively, non-calcified aortic valve morphology, typically observed in younger patients with conditions such as rheumatic AS or pure native AR, presents unique challenges for TAVI. In typical cases, baseline calcifications provide essential anchoring for the THV, and the absence of calcification increases the risk of valve embolization or dislocation during the procedure. SAVR offers a more controlled environment for addressing non-calcified aortic valves, allowing for the precise placement and secure fixation of the valve, thereby reducing the risk of complications associated with valve embolization or dislocation.

Thus, for patients with extreme (excessive or minimal) asymmetrical calcification patterns in the aortic annulus or LVOT, SAVR should be strongly considered as the preferred treatment, as it allows for direct visualization and precise surgical management of calcific deposits, and ensures more accurate valve seating.

### 2.4. Low Take-Off of Coronary Ostia and a Shallow Sinus of Valsalva

Coronary obstruction (CO) following TAVI is a serious procedural complication that carries a mortality rate of 50% [69,70]. Although its incidence is relatively low, CO remains a critical clinical concern when deciding between SAVR and TAVI for AS. The underlying mechanism of CO typically involves displacement of heavily calcified native valve leaflets or sinus sequestration caused by the THV, leading to coronary artery obstruction. Certain anatomical features have been identified as significant risk factors for CO during TAVI. A low coronary ostium take-off, defined as a coronary ostium height less than 12 mm—particularly below 10 mm—poses the greatest risk. Similarly, a shallow sinus of Valsalva, with a diameter less than 30 mm, further increases susceptibility to CO [70]. Other high-risk anatomical features include a cusp height exceeding the coronary ostium height, a low and narrow sinotubular junction (STJ height < 15 mm and diameter < 20 mm), a virtual transcatheter-to-coronary (VTC) distance of ≤4 mm [71,72], and extensive calcification of the native valve leaflets, with calcification volumes exceeding 600 mm³. Given these risks, patients with anatomical features such as low coronary ostium take-off and shallow sinuses of Valsalva are generally better suited for SAVR to mitigate the likelihood of TAVI-induced CO. Of note, patients who previously underwent a subcoronary approach for stentless valve implantation during SAVR, rather than full root replacement, have been associated with an increased risk of CO.

Nonetheless, for patients deemed high-risk for surgical interventions, addressing these anatomical challenges requires careful pre-procedural planning. Recent advancements in pre-procedural TAVI planning have introduced patient-specific computer simulations, which integrate deep learning and finite element analysis (FEA) to simulate interactions between devices and native anatomy [73]. These simulations improve decision-making by predicting device interactions with patient-specific anatomy, optimizing THV selection and positioning, and mitigating risks like PVL and PPI. Although clinical experience remains limited, early trials, like PRECISE TAVI [74], offer promising outcomes in complex anatomies. Moreover, some preventative strategies have been proposed during TAVI to mitigate risks of CO, such as coronary protection and the bioprosthetic aortic scallop intentional laceration (BASILICA) technique [75]. The BASILICA technique involves incising the aortic scallop to prevent iatrogenic CO and has demonstrated potential utility [76], although its efficacy in low-risk patients remains less well established [77]. It also faces limitations with newer-generation valves like Sapien 3 and Evolut, where narrower leaflet splits reduce its effectiveness [78]. Otherwise, prophylactic stenting of the affected coronary arteries can be considered; however, this approach is associated with a high risk of stent compression and thrombosis, particularly in cases where there is a risk of bioprosthetic valve leaflet displacement [79]. Further studies are needed assess the comparative efficacy of these preventative strategies, ensuring an adequate safety profile for TAVI in patients with low coronary ostia take-off and a shallow sinus of Valsalva anatomy.

### 2.5. Small Aortic Annulus

Furthermore, patients with a small aortic annulus (SAA)—generally defined as an aortic annulus measuring ≤23 mm or an annular area of <430 mm^2^ [80,81,82]—present limitations in THV positioning and anchoring during TAVI, increasing the risk of patient-prosthesis mismatch (PPM) [83,84,85]. While TAVI allows for the possibility of oversizing the THV to mitigate the risk of severe PPM, this approach has not been shown to significantly lower severe PPM rates [86,87], and these potential benefits are moreover outweighed by the increased risks of annular rupture and CO associated with oversizing. The recent SMART trial reported moderate or severe PPM at 30 days in 11.2% of patients with self-expanding valves (SEV) and 35.3% in the balloon-expandable valve (BEV) group, with incidences of hemodynamic structural valve dysfunction at 3.5% and 32.8%, respectively. The VIVA trial, comparing SAVR and TAVI in AS patients with SAA, found no significant difference in valve hemodynamic results and clinical outcomes after a median follow-up of 2 years [88]. Moreover, a post hoc analysis of women with SAA from the SURTAVI trial, comparing TAVI and SAVR, found similar clinical outcomes between the groups up to 2 years, with a lower incidence of new pacemaker implantation in the SAVR group. However, women in the TAVI group showed superior valve hemodynamics, including larger effective orifice areas (EOA), lower mean gradients, and less moderate or severe PPM. A recent meta-analysis (2024 [89] reported that TAVI was associated with significantly lower rates of 30-day and 2-year major bleeding and a reduced rate of moderate PPM. No significant difference was found between TAVI and SAVR regarding short-term mild AR and moderate/severe AR. While these findings suggest that both TAVI and SAVR are viable options for SAA patients with comparable efficacy in valve hemodynamics and clinical outcomes, SAVR offers distinct advantages for individuals requiring concurrent aortic root enlargement, which can reduce postoperative PPM by up to 50% [90], or the use of stentless valves to improve the effective orifice area. Notably, in the VIVA trial [88], about one-third of patients in the surgical group underwent aortic root enlargement or sutureless valve implantation. Additionally, SAVR is particularly beneficial for younger SAA patients who may require future valve replacement interventions. By enabling pre-emptive aortic root enlargement and valve replacement, SAVR effectively mitigates the risk of CO in potential valve-in-valve (ViV) procedures, addressing long-term management needs before patients become high-risk surgical candidates due to advanced age or comorbidities. Of note, a recent meta-analysis by Tanaka et al. (2024) [91] revealed that SAVR with ascending aortic enlargement is not associated with increased perioperative morbidity or mortality, although there is no conclusive indication on its benefits on mid-term survival, freedom from reoperation, and freedom from heart failure after SAVR. Hence, the decision between TAVI and SAVR for patients with SAA should be guided by a comprehensive assessment of annulus size, the need for concurrent root enlargement intervention, and lifetime considerations.

## 3. Concomitant Disease

SAVR allows for simultaneous interventions during aortic valve replacement and may be the preferred approach for patients with concomitant conditions requiring multiple procedures. Given the progressive nature of AS and its higher prevalence in the elderly population, patients with AS often present with various comorbidities. Current guidelines recommend SAVR with concomitant coronary artery bypass grafting (CABG) for AS patients with multivessel CAD [17], as studies indicate poorer outcomes following TAVI in patients with CAD compared to those without CAD [92,93,94] (Table 1). Moreover, surgery that includes concomitant atrial fibrillation ablation through a MAZE procedure may be appropriate for patients with chronic atrial fibrillation (AF). Evidence suggests that patients with severe AS and AF who undergo valve replacement without AF ablation experience worse long-term mortality outcomes [95].

Moreover, up to 30% of patients with severe symptomatic AS also have concurrent valvular diseases, such as mitral or tricuspid valve disease [96,97,98]. Mitral regurgitation (MR), present in 11.5–36.8% of TAVI recipients [35], is the most prevalent coexisting valvular condition in AS patients, often resulting from the elevated left ventricular pressure associated with AS [36]. Current guidelines recommend mitral valve surgery for patients with asymptomatic moderate-to-severe primary MR who are undergoing SAVR [17,37] (Table 1). In cases where patients with severe AS and mitral stenosis (MS) have unfavorable anatomy, such as a heavily calcified annulus and leaflets, concomitant surgical replacement of both the aortic and mitral valves is recommended [17,19,37]. For patients with isolated MS and favorable anatomy, a potential management strategy could involve TAVI followed by staged percutaneous mitral commissurotomy. Additionally, current guidelines recommend tricuspid valve surgery in patients undergoing SAVR who have severe tricuspid regurgitation (TR) (Class I indication) or moderate TR with a dilated annulus ( ≥40 mm) (Class IIa indication) [17,19] (Table 1). For patients deemed high-risk or inoperable, a transcatheter tricuspid valve intervention may be considered for persistent or worsening TR after TAVI [38,99].

Overall, SAVR remains the preferred treatment strategy for patients with multivessel disease or left main coronary artery involvement, severe mitral and/or tricuspid valve disease, or aortopathy exceeding 4.5 cm, except in cases where the surgical risk is high or prohibitive.

## 4. Young Patient and Lifetime Management

TAVI has recently emerged as a viable option for younger low-risk patients. However, there remains limited evidence regarding its long-term outcomes in this population, especially concerning valve durability, the risk of long-term complications, and the potential need for reintervention over a patient’s lifetime.

An important consideration for younger patients is the potential long-term impact on quality of life due to complications following SAVR or TAVI. While both procedures carry inherent risks, TAVI has been associated with a high incidence of long-term post-procedural complications. It is reported that over 41% of patients undergoing TAVI experience more than trace PVL, with moderate-to-severe PVL occurring in about 2% of cases [100]. While rates of moderate-to-severe PVL are comparable between TAVI and SAVR, mild PVL is significantly more frequent after TAVI (30–36%) compared to surgical valves (3%) [8,17]. Mild PVL can accumulate negative effects over time and is linked to increased all-cause mortality at five years post-TAVI, regardless of a patient’s baseline clinical or echocardiographic profile [19]. Additionally, conduction abnormalities requiring permanent pacemaker implantation (PPI) after TAVI remain an important concern, as they are associated with an elevated risk of mortality and rehospitalization, primarily due to secondary congestive heart failure [101]. Recent studies identified PPI and PVL following TAVI as independent predictors of mortality [8,12]. In the PARTNER_3_ and Evolut low-risk clinical trials, the 30-day risk of PPI after TAVI ranged from 6.5% to 24% at the longest follow-up, with SAVR showing lower rates [8,60,69,70,102] (Table 2). While these complications should not solely dictate treatment decisions for low-risk patients with AS, they are critical to consider.

**Table 2:** Long-Term Clinical Outcomes and Valve Durability in Low-Risk Patients Undergoing TAVI vs. SAVR: Evidence from Major Trials.

	NOTION [103] Thyregod et al. 2024	PARTNER 3 [104] Mack et al. 2023	Evolut Low Risk [12,105] Forrest et al. 2023 Popma/Reardon et al. 2019
**Follow-up**	10 years	5 years	4 years
**Number of patients**	145 (TAVI) 135 (SAVR)	503 (TAVI) 497 (SAVR)	725 (TAVI) 678 (SAVR)
**Age**	79.2 years (TAVI) 79.0 years (SAVR)	73.3 years (TAVI) 73.6 years (SAVR)	74.1 years (TAVI) 73.6 years (SAVR)
**Risk stratification**	STS score <4%: 83% (TAVI) STS score <4%: 80% (SAVR)	STS score: 1.9% (TAVI) STS score: 1.9% (SAVR) EuroSCORE II: 1.5% (TAVI) EuroSCORE II: 1.5% (SAVR)	STS score: 1.9% (TAVI) STS score: 1.9% (SAVR)
Primary Outcome *	Composite All-cause mortality, stroke, or MI 65.5% vs. 65.5% (*p* = 0.90)	Composite All-cause mortality, stroke, or rehospitalization ** 22.8% vs. 27.2% (*p* = 0.07)	Composite All-cause mortality or disabling stroke 10.7% vs. 14.1% (*p* = 0.050) All-cause mortality, disabling stroke, or aortic valve rehospitalization 18.0% vs. 22.4% (*p* = 0.04)
All-Cause Mortality *	62.7% vs. 64.0% (*p* = 0.80)	10.0% vs. 8.2% (*p* = 0.35)	9.0% vs. 12.1% (*p* = 0.07)
Stroke *	9.7% vs. 16.4% (*p* = 0.10)	5.8% vs. 6.4% (*p* = 0.60)	2.9% vs. 3.8% (*p* = 0.32)
Valve Durability *	SVD Severe—1.5% vs. 10.0% (*p* = 0.02) Moderate-or-greater—15.4% vs. 20.8% (*p* > 0.05) BVF 9.7% vs. 13.8% (*p* = 0.4) Valve reintervention 4.3% vs. 2.2% (*p* > 0.05)	BVF 3.3% vs. 3.8% (*p* > 0.05) Valve reintervention 2.6% vs. 3.0% (*p* > 0.05) Valve thrombosis 2.5% vs. 0.2% (*p* < 0.05)	Valve reintervention 1.3% vs. 1.7% at 4 yrs (*p* = 0.63)
Other Outcomes *	Pacemaker 44.7% vs. 14.0% (*p* < 0.01) PVL Moderate/severe at 5 yrs—8.2% vs. 0% (*p* < 0.001) Endocarditis 7.2% vs. 7.4% (*p* = 1.0)	New-onset Afib 13.7% vs. 42.4% (*p* < 0.05) Major bleeding 10.2% vs. 14.8% (*p* < 0.05) Pacemaker 6.5% vs. 4.0% at 30 days (*p* > 0.05) Paravalvular leak Mild-or-greater—20.8% vs. 3.2% (*p* < 0.001) Moderate/severe—0.9% vs. 0% (*p* > 0.05) Rehospitalization ** 13.7% vs. 17.4% (*p* = 0.09)	Permanent pacemaker 24.6% vs. 9.9% (*p* < 0.001) Paravalvular leak No/trace—84.7% vs. 98.4% (*p* < 0.05) Moderate-or-greater—0.4% vs. 0.0% (*p* = 0.50) Valve endocarditis 0.9% vs. 2.2% (*p* = 0.06)

* Results presented as TAVI vs. SAVR; ** Rehospitalization related to heart failure or valve-related rehospitalization. Abbreviations: TAVI—Transcatheter Aortic Valve Implantation; SAVR—Surgical Aortic Valve Replacement; STS—Society of Thoracic Surgeons; MI—Myocardial Infarction; SVD—Structural Valve Deterioration; BVF—Bioprosthetic Valve Failure; PVL—Paravalvular Leak; Afib—Atrial Fibrillation.

Moreover, age has been established as a significant predictor of valve longevity, with the risk of structural valve deterioration (SVD) significantly increasing in those who undergo valve implantation at a younger age [106]. Indeed, younger patients experience accelerated structural deterioration, increasing the risk of long-term complications and the need for reintervention over time. Therefore, valve durability following TAVI or SAVR is a critical factor in determining the optimal treatment strategy for younger patients to ensure effective lifetime management. Bioprosthetic valves are the most used in both surgical and transcatheter valve replacements, given their lower thrombogenicity and benefit of not requiring long-term anticoagulation; however, bioprotheses are notably susceptible to SVD. Long-term durability studies of bioprosthetic surgical valves have primarily relied on reintervention rates, which likely underestimate the true incidence of SVD [107,108] (Table 2). Reported reintervention rates are relatively low, with <7% at 10 years and <15% at 20 years, though substantial variation exists based on patient age, as well as the design, generation, and model of the implanted bioprosthetic valve [107]. Nonetheless, there remains limited evidence on the durability of TAVI beyond 5 years. The NOTION trial [109], which provides the longest reported follow-up of a patient population randomized to TAVI or SAVR at 10-years follow-up, revealed that TAVI was associated with significantly lower rates of severe structural valve deterioration (SVD: 1.5% vs. 10.0%, *p* < 0.05) and severe bioprosthetic valve dysfunction (BVD: 20.5% vs. 43.0%, *p* < 0.05) compared to SAVR, with no significant differences in bioprosthetic valve failure (BVF: 9.7% vs. 13.8%, *p* > 0.05) or aortic valve reintervention (4.3% vs. 2.2%, *p* > 0.05) [103]. While these results suggest a higher rate of SVD with SAVR, their interpretation is limited by the scarcity of long-term data on TAVI durability and the significant survivorship bias in the NOTION trial, where only 25% of patients were alive at the 10-year follow-up [103]. Additionally, the lack of standardization in reported definitions of SVD complicates drawing conclusive comparisons between the two approaches [110]. As evidence on TAVI durability beyond 5–10 years remains limited, guidelines continue to recommend SAVR with mechanical valves for patients with no contraindications to anticoagulation under 50–60 years of age [111]. This recommendation is supported by long-term data which demonstrates improved survival over a 15-year follow-up and reduced need for lifetime reintervention in patients under 55 who received mechanical valves compared to those with bioprosthetic valves [112,113,114]. Interestingly, the survival advantage of mechanical valves appears to be age-dependent [115]; patients aged 50–70 benefit from significantly better survival with mechanical valves, whereas bioprosthetic valves offer superior outcomes in patients over 70 years old. A recent meta-analysis by Warraich et al. (2024) [116] further highlights the benefits of mechanical valves, showing better overall survival, reduced risk of all-cause mortality, lower reoperation rates, and a decreased incidence of major bleeding compared to bioprosthetic valves in patients under 50 years old. However, while findings on the risk of stroke between mechanical and bioprosthetic valves are inconclusive across studies [115,117,118], there is consistent evidence that mechanical valves are associated with a higher incidence of bleeding compared to bioprosthetic valves. Ultimately, mechanical valves remain the preferred option for managing AS for younger patients without contraindications to anticoagulation due to their superior long-term outcomes in survival, reoperation rates, and stroke incidence.

Additionally, young patients with a life expectancy exceeding 20–25 years and suitable aorto–pulmonary anatomy may be eligible for the ROSS procedure. The ROSS procedure involves replacing a diseased aortic valve with the patient’s own pulmonary valve (i.e., pulmonary autograft), followed by the replacement of the pulmonary valve with a pulmonary homograft. The ROSS procedure was traditionally intended for pediatric cases with congenital abnormalities; however, it has since proven effective across various age groups, including select elderly patients. Recent meta-analyses confirmed that the ROSS procedure provides excellent long-term survival in both pediatric (pooled 10-year survival rate of 91.1% [119]) and adult populations (97.6% at 10 years and 87.4% at 20 years [97]). Additionally, a significant benefit of the Ross procedure is its ability to circumvent the necessity of lifelong anticoagulation therapy associated with mechanical aortic valve replacement, while yielding comparable or even superior clinical outcomes. A meta-analysis by Pompeu Sá et al. (2024) [120] revealed that patients who underwent the ROSS procedure had a significantly lower risk of mortality and lower risk of stroke and endocarditis [121] compared to patients who received a mechanical valve or a bioprosthetic valve at 5- and 15-years post-procedure. However, both the pulmonary homograft and the pulmonary autograft are susceptible to valve degeneration over time, with 20% of patients requiring reintervention within 20 years. While its durability significantly surpasses that of bioprosthetic valve replacements, thereby reducing the likelihood of multiple aortic reinterventions over a patient’s lifetime, the cumulative risk of reoperation was higher for the ROSS procedure compared to mechanical valves [120]. Hence, the Ross procedure presents a valuable option for select young patients with suitable aorto–pulmonary anatomy, offering favorable long-term survival outcomes, a lower risk for reintervention, and avoidance of anticoagulation.

**Figure 3:**
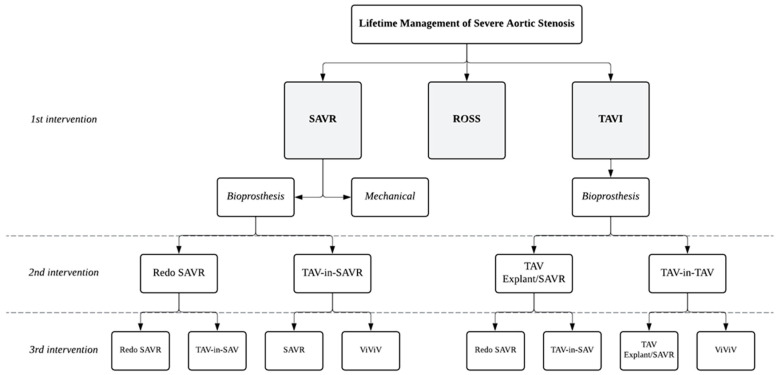
Lifetime Management Strategies for Severe Aortic Stenosis: Sequential Intervention pathways. Abbreviations: SAVR—Surgical Aortic Valve Replacement, TAVI—Transcatheter Aortic Valve Implantation, ViViV—Valve-in-Valve-in-Valve.

Nevertheless, whether younger patients undergo SAVR or TAVI for their first intervention, they will most likely require additional interventions during their lifetime. The initial intervention has significant procedural implications on future procedures, and therefore, healthcare teams must shift their therapeutic approach to consider the potential sequence of interventions over a patient’s lifetime, a concept known as “lifetime management” of aortic valve disease (Figure 3). While multiple open-heart surgeries are generally not ideal for most patients, integrating SAVR remains a crucial component of lifetime considerations for multiple reinterventions, as it mitigates the cumulative risks associated with ViV procedures, such as CO, and enables the management of concomitant diseases before patients become higher-risk due to comorbidities.

Redo SAVR and TAV-in-SAV are both available options for a second surgical intervention following an initial SAVR procedure. While long-term data for both approaches remain limited, short-term outcomes at 30-day, including mortality, stroke, major bleeding, and length of hospital stay, suggest TAV-in-SAV to be superior to redo SAVR [122]. Prior cardiac surgery is a significant risk factor for complications around redo-SAVR, primarily due to scar tissue formation and adhesions from the previous surgery. However, redo SAVR tends to have lower risks for CO and PPM [8,60] compared to TAV-in-SAV. Indeed, while TAV-in-SAV is the preferred treatment for older patients with reasonable risk stratification for PVL and PPI, special considerations are necessary for younger patients who may require a third intervention in the future. In cases where TAV-in-SAV fails, a subsequent ViV therapy (TAV-in-TAV-in-SAV) might be feasible, particularly for patients with larger aortic roots; however, the cumulative risk of CO increases with each ViV procedure. Redo SAVR provides the advantage of preserving the option for TAV-in-SAV as a third intervention when the patient is older and may be less able to tolerate open-heart surgery, with a reduced risk of CO at that time. In summary, TAV-in-SAV is generally preferred for SVD in older patients, while redo SAVR remains appropriate for younger patients who may require future interventions or have developed unfavorable anatomy for ViV procedures that require additional surgical adjustments.

For the third valve replacement, a hybrid strategy that combines surgical and transcatheter approaches (i.e., TAVI-SAVR-TAVI or SAVR-TAVI-TAVI) could be perceived as a potential solution, limiting the need for multiple surgeries over a patient’s lifetime. Although scenarios like SAVR-SAVR-TAVI could still be considered viable, it is important to avoid sequences like TAVI-TAVI-SAVR, where a final surgical procedure becomes more complex as the patient ages and accumulates comorbidities. In such cases, the increased complexity of surgery at an advanced age, along with the associated risks, makes this sequence less favorable. However, it is important to note that there are no data to support these specific strategies, and their effectiveness and appropriateness remains speculative. Ultimately, treatment strategies should be tailored to individual patient profiles, with careful consideration of the patient’s age, lifestyle, comorbidities, and long-term outlook, along with procedural risks and outcomes.

## 5. Infective Endocarditis

Native valve endocarditis (NVE) is an absolute contra-indication for TAVI and surgical treatment remains the gold standard [123,124]. Prosthetic valve infective endocarditis (PVE) is a life-threatening infection that affects both the valve replacement prosthesis and surrounding tissue, often following bacteremia. It can occur after both SAVR and TAVI, with an incidence ranging from 0.4 to 1.9 cases per 100 patient-years, and a mortality rate between 23% and 52% [125,126]. Importantly, studies have found no significant difference in the incidence or timing of PVE between SAVR and TAVI patients. However, treatment strategies remain largely based on general guidelines for infective endocarditis (IE), as no randomized trials have been conducted to compare different approaches specifically for PVE [124]. In addition to PVE, infective endocarditis can also occur on native heart valves. Conservative management with antibiotics is a common first-line treatment for both PVE and native valve endocarditis. However, in cases of severe complications—such as valve destruction, regurgitation, or obstruction from large vegetations—surgical intervention becomes necessary. Surgery is crucial when infective endocarditis leads to structural damage such as valve dehiscence, perforation, rupture, fistulas, or the development of large perivalvular abscesses [124]. Infection extending into the myocardium, especially if accompanied by new atrioventricular block, significantly increases the risk of mortality. Surgical intervention is often associated with better outcomes in these high-risk patients, particularly if performed early, compared to medical therapy alone [126]. However, surgery is still underutilized in many cases, despite evidence suggesting that a more aggressive surgical approach may improve survival. For patients with intermediate to low surgical risk, surgery can be highly beneficial, regardless of whether the infection originated from a TAVI, SAVR, or native valve. In summary, while antibiotics play a role in managing both PVE and NVE, surgery is often necessary for patients with severe complications. Early and aggressive surgical intervention tends to improve outcomes, particularly when infection leads to extensive tissue damage or poses an increased risk of mortality. Further research is needed to refine the timing and indications for surgery, especially in patients with TAVI-related PVE, as well as to better understand the management of NVE when surgery is indicated.

## 6. Tavi Explant

TAVI explantation is a technically demanding and high-risk procedure typically reserved for cases of severe complications, including PVE (43.1%), SVD (20.1%), PVL (18.2%), or patient–prosthesis mismatch (10.8%) [127]. The presence of neo-endothelialization around the prosthesis, particularly in valves implanted for more than one year, often necessitates complex surgical techniques such as aortic endarterectomy or aortic root replacement. These factors contribute to the elevated mortality risk associated with TAVI explantation compared to alternative approaches. Data from the Explant-TAVR registry [127,128] reported a one-year mortality rate of 28.5% following TAVI explantation. Findings from the EXPLANTORREDO-TAVR registry [129] reported significantly higher 30-day (13.6% vs. 3.4%, *p* < 0.001) and one-year (32.4% vs. 15.4%, *p* = 0.001) mortality rates for TAVI explantation compared to TAV-in-TAV. Interestingly, a landmark analysis [129] from the same registry showed higher 30-day mortality for TAVI explantation (*p* < 0.001), but comparable rates beyond 30 days (*p* = 0.91) with similar actuarial estimates at three years (30.4% vs. 27.1%). Additionally, a patient-matched analysis comparing TAV-in-TAV to TAVI explantation corroborated these findings with significantly higher 30-day mortality for TAVI explantation compared to TAV-in-TAV (12.3% vs. 6.2%, *p* = 0.050); however, one-year mortality rates converged between the two approaches (20.8% vs. 21.0%, *p* = 1.0) [130]. The choice of surgical strategy is further influenced by the type of prosthesis used during the initial TAVI procedure, which can complicate reinterventions requiring extensive aortic reconstruction. This complexity is further heightened by newer-generation valves, which feature taller sealing skirts and high-frame designs [78]. These characteristics, while improving sealing and durability, may exacerbate the challenges of TAVI explantation, particularly in cases requiring extensive aortic reconstruction [128]. Consequently, patient selection and prosthesis design remain critical considerations when planning reintervention strategies.

While TAV-in-TAV serves as a less invasive alternative to TAVI explantation [131,132,133,134,135], it remains contraindicated in specific clinical scenarios due to anatomical, procedural, and pathological challenges. A small annular diameter is a significant limitation, as it predisposes patients to PPM, resulting in elevated transvalvular gradients and compromised hemodynamic performance. The use of supra-annular devices and bioprosthetic valve fracture techniques are effective strategies for achieving an appropriate low gradient; however, TAV explant with SAVR, along with specialized techniques for aortic root enlargement or replacement (i.e., Bentall procedure) presents a viable option [136,137]. High risk of CO also precludes TAV-in-TAV, with risks influenced by reduced neo-sinuses, leaflet height, and tall valve frames. Tang et al. [138] developed a classification system to stratify coronary obstruction risk, identifying TAV-in-TAV as unfeasible when the valve-to-sinus height or distance is less than 2 mm.

Techniques such as the chimney approach and BASILICA aim to mitigate this risk. The chimney technique, though effective in valve-in-valve settings, is technically challenging in TAV-in-TAV due to stent placement between the layers of THvs. and questions about long-term stent patency. BASILICA has gained attention as a potential solution, with case reports, including Damlin et al. [139], demonstrating successful outcomes in select patients. However, feasibility is limited by valve type, anatomy, and alignment. Khan et al. [140] conducted an in vitro assessment of the feasibility of BASILICA in TAV-in-TAV procedures and observed that the leaflets of the index THV often failed to split adequately and were unable to extend beyond the confines of the THV frame. Additionally, commissural alignment during the initial TAVI is crucial for optimizing subsequent TAV-in-TAV outcomes;, yet this is not consistently achieved even with platforms like Evolut FX (>90% alignment success) [138]. In extreme cases where TAV-in-TAV poses a high risk of CO, hybrid surgical approaches such as SURPLUS TAVR may be necessary [141]. This involves a trans-aortic approach with direct leaflet resection under extracorporeal circulation, enabling precise valve implantation and commissural alignment. The risk of stroke following TAV-in-TAV also warrants close attention. Current data report stroke rates of 0.5–3% at 30 days [142], comparable to native TAVI populations. A meta-analysis (2021) [143] indicates no significant difference in stroke incidence between TAV-in-SAVR and native TAVI. Preventive strategies, such as embolic cerebral protection devices, remain controversial, with no consistent reduction in stroke rates demonstrated [144]. These devices may still have utility in high-risk patients, such as those with extensive leaflet calcifications or a history of valve thrombosis. Further studies are needed to define their role in TAV-in-TAV populations. In conclusion, while TAV-in-TAV presents a valuable alternative for managing complex cases, its application is constrained by significant anatomical and procedural challenges. Surgical TAVI explantation remains a cornerstone in the management of these patients, underscoring the importance of meticulous preoperative assessment, comprehensive planning, and thorough risk evaluation to optimize outcomes.

## 7. Conclusions

TAVI has seen growing adoption across the entire surgical risk spectrum, demonstrating efficacy and safety in a wide range of patients. However, it remains limited in certain populations, particularly those with complex anatomies, bicuspid aortic valves, pure aortic regurgitation, and younger individuals who require lifetime valve management. In these cases, SAVR continues to be a crucial intervention, offering more durable solutions and the ability to address concurrent surgical needs. In conclusion, while TAVI has significantly transformed the treatment landscape for aortic valve diseases, SAVR remains indispensable for select patient groups. The choice between TAVI and SAVR should be carefully individualized based on patient characteristics, ensuring the best possible long-term outcomes. 

## Figures and Tables

**Table 1:** Comprehensive Summary of Treatment Indications between TAVI and SAVR: Evidence-Based Strategies and Guideline Recommendations by Subgroup Population.

Patient Population	Preferred Treatment Strategy	Key Clinical Trial Evidence	Guideline Recommendations *^,‡^
**High-Risk or Inoperable Severe AS** (STS >8%)	**TAVI preferred** (transfemoral); SAVR not an option	PARTNER 1B CoreValve High-Risk Trial	ACC/AHA: TAVI (Class I) ESC/EACTS: TAVI if ≥75 years or STS >8% (Class I)
**Intermediate-Risk Severe AS** (STS 4–8%)	**TAVI or SAVR**; Heart Team decision based on anatomy and comorbidities	PARTNER 2A SURTAVI	ACC/AHA & ESC/EACTS: Shared decision-making by Heart Team
**Low-Risk Severe AS** (STS <4%)	**SAVR** (<65 years); TAVI for older patients	PARTNER 3 Evolut Low Risk NOTION	ACC/AHA: <65—SAVR (Class I) 65–80—SAVR/TAVI (Class I) >80—TAVI (Class I)/SAVR (Class II) ESC/EACTS: SAVR (Class I)
**Bicuspid Aortic Valve** (BAV)	**SAVR preferred** for young/low-risk or with aortopathy; TAVI selectively (older/high-risk)	NOTION-2 PARTNER 3 BAV Registry	ACC/AHA & ESC/EACTS: SAVR preferred in low-risk BAV or those with aortopathy; TAVI selectively
**Severe Aortic Regurgitation** (AR)	**SAVR preferred**; TAVI if surgery is prohibitive	ALIGN-AR PANTHEON	ACC/AHA & ESC/EACTS: SAVR in symptomatic AR or reduced LVEF (Class I)
**Small Aortic Annulus** (SAA)	**TAVI** (supra-annular valves) or **SAVR** with annular enlargement	SMART VIVA SURTAVI	ACC/AHA: consider surgical annulus-enlarging procedures. ESC/EACTS: consider aortic valve-sparing root replacement for younger patients with an enlarged aortic root and normal cusp motion
**Young Patients with AS** (≤65 years)	**SAVR preferred** (mechanical valve or Ross procedure)	NOTION-2 Ross Procedure studies	ACC/AHA & ESC/EACTS: SAVR preferred in patients <65 or with >20-year life expectancy
**Concomitant Surgical Needs**	**SAVR** with combined procedures; TAVI only high-risk	Observational studies FREEDOM trial data	ACC/AHA & ESC/EACTS: Class I for SAVR in AS with multivessel CAD (CABG required), severe mitral/tricuspid disease, or aortopathy >4.5–5.0 cm.
**Infective Endocarditis**	**SAVR preferred**	Observational studies	ACC/AHA & ESC: Class I for SAVR in infective endocarditis; TAVI not recommended

* ACC/AHA: 2020 Guidelines of the American College of Cardiology/American Heart Association for the Management of Patients with Valvular Heart Disease. ^‡^ ESC/EACTS: 2021 Guidelines of the European Society of Cardiology/European Association for Cardio-Thoracic Surgery.

## Abbreviations

AS	Aortic Stenosis
AR	Aortic Regurgitation
MS	Mitral Stenosis
TR	Tricuspid Regurgitation
SAVR	Surgical Aortic Valve Replacement
TAVI	Transcatheter Aortic Valve Implantation
BAV	Bicuspid Aortic Valve
SAA	Small Aortic Annulus
THV	Transcatheter Heart Valve
LVOT	Left Ventricular Outflow Tract
PVL	Perivalvular Leakage
PPI	Permanent Pacemaker Implantation
VSRR	Valve-Sparing Aortic Root Replacement
AVC	Aortic Valve Calcification
CO	Coronary Obstruction
STJ	Sinotubular Junction
VTC	Virtual Transcatheter-to-Coronary
FEA	Finite Element Analysis
CEPD	Cerebral Embolic Protection Devices
PPM	Patient–Prosthesis Mismatch
EOA	Effective Orifice Areas
ViV	Valve-in-Valve
CAD	Coronary Artery Disease
CABG	Coronary Artery Bypass Grafting
AF	Atrial Fibrillation
NVE	Native Valve Endocarditis
PVE	Prosthetic Valve Infective Endocarditis
IE	Infective Endocarditis
SVD	Structural Valve Deterioration
BVD	Bioprosthetic Valve Dysfunction
BVF	Bioprosthetic Valve Failure
MI	Myocardial Infarction
STS	Society of Thoracic Surgeons
ESC	European Society of Cardiology
EACTS	European Association for Cardio-Thoracic Surgery
ACC	American College of Cardiology
AHA	American Heart Association
